# The Mediating Effect of Reassurance-Seeking Behavior on the Influence of Viral Anxiety and Depression on COVID-19 Obsession Among Medical Students

**DOI:** 10.3389/fpsyt.2022.899266

**Published:** 2022-06-13

**Authors:** Hyun Sub Kim, Junseok Ahn, Jukab Lee, Youjin Hong, Changnam Kim, Jangho Park, Seockhoon Chung

**Affiliations:** ^1^Department of Psychiatry, Ulsan University Hospital, University of Ulsan College of Medicine, Ulsan, South Korea; ^2^Department of Psychiatry, GangNeung Asan Hospital, University of Ulsan College of Medicine, Gangneung, South Korea; ^3^Department of Psychiatry, Samsung Changwon Hospital, Sungkyunkwan University of Medicine, Changwon, South Korea; ^4^Department of Psychiatry, Asan Medical Center, University of Ulsan College of Medicine, Seoul, South Korea

**Keywords:** COVID-19, medical students, obsession, reassurance-seeking behavior, SAVE-6

## Abstract

**Objectives:**

Healthcare workers experienced great psychological burden due to the continuation of the COVID-19 pandemic. During the pandemic, medical healthcare workers experienced greater instances of insomnia, anxiety, depression, somatization, and obsessive-compulsive symptoms than the general population. This study aimed to explore the association between viral anxiety and obsession with COVID-19 among medical students with reassurance-seeking behavior as a mediator.

**Methods:**

In October 2021, an online survey was conducted among medical students at the University of Ulsan College of Medicine. The clinical characteristics of 162 participants and their responses to rating scales, including stress and anxiety to viral Epidemics-6 items (SAVE-6), Coronavirus Reassurance-Seeking Behaviors Scale (CRBS), Patient Health Questionnaire-9 items (PHQ-9), and Obsession with COVID-19 scale were collected.

**Results:**

Medical students' obsession with COVID-19 was based on the PHQ-9 (β = 0.15, *p* = 0.01), SAVE-6 (β = 0.43, *p* < 0.001), and CRBS (β = 0.38, *p* < 0.001) scores (adjusted R^2^ = 0.49, *F* = 39.9, *p* < 0.001). Mediation analysis showed that medical students' viral anxiety and depression directly influenced their obsession with COVID-19, and their reassurance-seeking behavior partially mediated the effects of depression or viral anxiety on obsession with COVID-19.

**Conclusions:**

Medical students' viral anxiety and depression may affect their obsession with COVID-19, and reassurance-seeking behaviors may mediate this. Therefore, medical students should adopt adaptive coping strategies to prevent high levels of viral anxiety and recurrent reassurance-seeking behavior.

## Introduction

As of January 02, 2022, the global cumulative number of SARS-CoV-2 cases has exceeded 289 million (1). Despite administrative orders, including those of vaccinations and stay-at-home, millions of new cases are reported every week, and most new cases are caused by the Delta variant. In South Korea, 583,065 people were confirmed COVID positive, and 4,906 died during the 2 years of the pandemic (2, 3). By the end of 2021, 82% of people in South Korea had been vaccinated (2) and have practiced COVID-19 safety measures. The COVID-19 pandemic has brought about many changes, and psychological distress is one of them. A survey in China investigated the association between mental health symptoms, including depression and anxiety, their prevalence and risk factors during the COVID-19 pandemic. In this study, depressive and anxiety symptoms were reported in 27.9, and 31.6% of the participants, respectively. Additionally, those with confirmed or suspected COVID-19, those at risk of occupational exposure, and residents of Hubei Province accounted for more severe symptoms (4). In Spain, as a result of surveying the degree of depression and anxiety among the general population during the COVID-19 pandemic through an online survey, 27.5% of participants reported severe depression and 20.8% reported severe anxiety (5). The prevalence of severe somatic complaints, anxiety, and depression during study periods, among college students in the Czech Republic, was 10.1, 4.9, and 3.4%, respectively (6).

### Psychological Distress Among Medical Students During COVID-19

In particular, frontline healthcare workers experienced noteworthy psychological effects during the COVID-19 outbreak (7, 8). A Chinese population study found that medical healthcare workers were more likely to report insomnia (38.4 vs. 30.5%), anxiety (13.0 vs. 8.5%), depression (12.2 vs. 9.5%), somatization (1.6 vs. 0.4%), and obsessive-compulsive symptoms (5.3 vs. 2.2%) than the general population (9). Possible reasons could be that healthcare workers play a major role in preventing the spread of the virus — taking care of infected or suspected patients while working in an environment that lacks protective equipment or is categorized by work overload (10, 11). In addition to fearing for their personal health, they might also be worried about infecting their families, friends, and colleagues, perceived stigmatization, or being scrutinized (12).

Additionally, healthcare workers played the role of healthcare workers in some cases (13). They had to step forward as frontline medical personnel in situations where they were not fully prepared to help prevent the spread of infectious diseases. Medical students usually study close to healthcare facilities and face massive potential dangers of contracting infectious diseases from patients during their clinical clerkship. They worry about transmitting the virus to their families and friends, increasing their anxiety and stress (14). To help provide some respite to medical students, most medical schools in Korea conducted online classes, despite their unfamiliarity with online teaching systems.

### Depression, Viral Anxiety, Reassurance-Seeking Behavior, and Preoccupation With COVID-19

The incidence of obsessive-compulsive disorder-related symptoms has increased since the COVID-19 pandemic (15, 16). This exacerbation of clinical symptoms was severe in patients with a prior diagnosis of obsessive-compulsive disorder or contamination with the symptom dimension of obsessive-compulsive disorder. Most symptoms were related to fear of contamination and excessive self-diagnosis for COVID-related symptoms (17). In addition, the characteristics of individuals who follow safety precautions, such as wearing a mask and washing hands, are significantly related to the increase in obsessive-compulsive symptoms and fear related to contamination (18).

Based on the model of hypochondriasis (currently, illness anxiety disorder), the fear of being sick influences one's anxiety, and may cause one to avoid the problem or seek reassurance. Paradoxically, recurrent engagement in reassurance-seeking behaviors may increase anxiety levels. As this process continues, it induces preoccupation with illness (19). If one has fears of being infected with COVID-19, he or she may pursue reassurance-seeking behavior, such as checking bodily sensations, hand washing, or repeated media search. Since people who have severe concerns about their health may try to seek reassurance, reassurance-seeking behavior can be a sign of preoccupation with the illness (20, 21).

Excessive reassurance-seeking behavior was first studied in the context of depression (22–24). It is a risk factor for depressive symptoms (24). Depression with reassurance-seeking behavior may lead to interpersonal problems, such as loneliness or devaluation as per Coyne's interpersonal theory of depression (22). Depression is also closely related to symptoms of anxiety, and this association has proven its existence with viral anxiety and depression during the COVID-19 pandemic among healthcare workers (8, 25). Depending on how individual college students perceive the stress related to the pandemic, it could manifest as anxiety, depression, or behavioral problems (26). Therefore, we speculate that depression or viral anxiety during this pandemic may provoke reassurance-seeking behavior, which may enhance preoccupation with COVID-19. In particular, medical students may amplify their reassurance-seeking behaviors.

In the present study, we would like to evaluate depression, viral anxiety, and obsessive thinking over the coronavirus among medical students due to various conditions that changed during the pandemic, and how reassurance-seeking behavior acts as a protective behavior against the COVID-19 pandemic during psychological difficulties. This study aims to explore the association between viral anxiety and reassurance-seeking behavior or obsession with COVID-19 among medical students. In addition, we explored whether reassurance-seeking behavior could mediate the association between viral anxiety and obsession with COVID-19. We hypothesized that (a) depression will be positively related to obsession with COVID-19, (b) viral anxiety will be positively associated with obsession with COVID-19, and (c) reassurance-seeking behavior will at least partially mediate the relationships between depression/viral anxiety and obsession with COVID-19 among medical students.

## Methods

### Participants and Procedure

From October 20–28, 2021, an online survey was conducted among medical students at the University of Ulsan College of Medicine (UUCM). We developed a survey form and posted an enrollment flyer on the notice boards of each lecture room for all medical students (*N* = 251) studying at UUCM. A total of 162 students voluntarily participated in this survey. Volunteers who selected “yes” on the question about “Do you agree that your response will be used for research purposes?” in the first part of the survey form were selected as participants for the study rather than obtaining written informed consent. The survey was conducted anonymously and personal information was not collected. We provided a gift card valued at USD $3 to each participant for participation. Sample size estimation was done based on the target population and all 251 medical students studying at UUCM. Out of 251 students, 162 responded to this survey. The study protocol was approved by the Institutional Review Board (IRB) of the Asan Medical Center (2021–1438) who waived the requirement for written informed consent.

The survey form, developed via Google Forms, included questions on students' age, gender, grades, and responses to COVID-19 questions, such as “Did you experience being quarantined due to infection with COVID-19?”, “Did you experience being infected with COVID-19?” or “Did you get vaccinated?'. Their past psychiatric history was checked by the question, “Have you ever experienced depression, anxiety, or insomnia or had treatment for it?”. Psychiatric distress was checked by the question, “Now, do you think you are depressed or anxious, or do you need help for your mood state?”. The survey form was developed according to the Checklist for Reporting Results of Internet e-Survey (CHERRIES) guidelines (27). After the development of an e-survey, the usability and technical functionalities were tested by investigators (SC) before implementation.

### Measures

#### Stress and Anxiety to Viral Epidemics-6 Items

The SAVE-6 is a rating scale that measures anxiety responses to viral epidemics (28). It is derived from the SAVE-9 scale which was originally developed for measuring healthcare workers' work-related stress and anxiety responses to viral epidemics (29). It consists of 6 items, each of which can be rated from zero (never) to four (always). The total score reflects the severity of anxiety response to a viral epidemic. The cut-off score was 15 for a mild degree of anxiety. In this study, we applied the original Korean version of the SAVE-6, and Cronbach's alpha was 0.782.

#### Coronavirus Reassurance-Seeking Behaviors Scale

The CRBS is a self-report measure of reassurance-seeking behaviors related to concerns about the coronavirus infection (30). The CRBS measures the excessive reassurance-seeking behavior of people who engage in coronavirus-related information. Each of the five items are rated on a scale of zero (not at all) to four (nearly every day). Although cut-off score has not yet been determined, the original developer suggested CRBS total scores ≥ 12 was above average reassurance-seeking activity. In this study, we applied the Korean version of the CRBS, which has not been formally validated. In this sample, the Kaiser–Meyer–Olkin measure was 0.768, and Bartlett's sphericity was *p* < 0.001. Confirmatory factor analysis for the single structure model of CRBS revealed a good model fit: Comparative Fit Index (CFI) of 1.000, Tucker-Lewis Index (TLI) of 1.086, Root Mean Square Error of Approximation (RMSEA) of 0.000, and Standardized Root Mean Square Residual (SRMR) of 0.031. The CRBS has good reliability, based on Cronbach's alpha (0.723) and McDonald's Omega (0.754).

#### Patient Health Questionnaire-9 Items

The PHQ-9 is a self-report rating scale with wide clinical usability, simplicity, and clear validity for measuring depression severity (31, 32). The nine items in this scale can be rated from zero (not at all) to three (nearly every day). In this study, we applied the Korean version of the PHQ-9(33, 34), and Cronbach's alpha was 0.845.

#### Obsession With COVID-19 Scale

The Obsession with COVID-19 Scale (OCS) is a self-report mental health screener that measures persistent disturbed thinking related to COVID-19 (35). It also helps identify individuals who are functionally impaired by COVID-19 related thinking patterns. The OCS has four items, and each item can be rated on a 5-point scale from 0 (not at all) to 4 (nearly every day over the last 2 weeks). The original developer suggested that OCS total score ≥7 indicates probable dysfunctional thinking about COVID-19. In this study, we used the Korean version of the OCS (36), and the Cronbach's alpha in this sample was 0.689.

## Statistical Analysis

Participants' demographic characteristics and rating scale scores are summarized as mean ± standard deviation. The level of significance for the analyses was defined as two-tailed at values of *p* < 0.05. The Kolmogorov-Smirnov normality test was done to examine whether variables are normally distributed or not, and we found that CRBS, OCD, and PHQ-9 scales scores did not distribute normally. Correlation analysis was performed using Spearman's correlation analysis. Linear regression analysis was performed to examine the variables that could predict the occurrence of COVID-19. To explore whether reassurance-seeking behavior mediates the influence of viral anxiety or depression on obsession with COVID-19, a bootstrap method with 2,000 re-samples was used.

Furthermore, we explored the validity and reliability of the Korean version of the CRBS (*Supplement data*) using this sample. Data suitability and sampling adequacy were assessed using the Kaiser-Meyer-Olkin (KMO) value and Bartlett's test of sphericity. In the CFA, model fit was assessed using CFI, TLI, RMSEA, and SRMR values (37, 38). We used SPSS version 21.0, AMOS version 27 for Windows (IBM Corp., Armonk, NY, USA), and JASP version 0.14.1 to perform the statistical analysis.

## Results

All 162 (64.5%) medical students out of 251 students, approximately 50–75% of students in each grade, at the Ulsan College of Medicine participated in this study. No responses were excluded for any participants. Among the participants, 71.6% were male, 21.7% were quarantined, 1.9% were infected, and all were vaccinated (Table 1). Fourteen point two percentage of students reported that they had experienced or been treated for depression, anxiety, or insomnia, and 5.6% reported that they were depressed or anxious or needed help for their mood state. The mean PHQ-9, SAVE-6, CRBS, and OCS scores were 3.6, 9.2, 2.0, and 1.7, respectively, which were below the cut-off values for depression, viral anxiety, and obsession with COVID-19. The participants' rating scale scores are presented in Table 1.

**Table 1:** Clinical characteristics of participants (*N* = 162).

**Variables**	***N* (%) Mean ±SD**
**Sex (male)**	116 (71.6%)
**Age, years old**	22.3 ± 2.1
**Grades**	
Pre-medicine 1st (UUCM, total *N* = 43)	27 (62.8%)
Pre-medicine 2nd (UUCM, total *N* = 44)	27 (61.4%)
Medicine 1st (UUCM, total *N* = 44)	23 (52.3%)
Medicine 2nd (UUCM, total *N* = 39)	29 (74.4%)
Medicine 3rd (UUCM, total *N* = 40)	30 (75.0%)
Medicine 4th (UUCM, total *N* = 41)	26 (63.4%)
**Questions on COVID-19**	
Did you experience being quarantined due to infection with COVID-19? (Yes)	35 (21.7%)
Did you experience being infected with COVID-19? (Yes)	3 (1.9%)
Did you get vaccinated? (Yes)	162 (100.0%)
**Psychiatric history**	
Have you ever experienced depression, anxiety, or insomnia or had treatment for it? (Yes)	23 (14.2%)
Now, do you think you are depressed or anxious, or do you need help for your mood state? (Yes)	9 (5.6%)
**Rating scales**	
Patient health questionnaire-9 items	3.6 ± 3.6
Stress and anxiety to viral epidemics-6 items	9.2 ± 4.5
Coronavirus reassurance-seeking behaviors scale	2.0 ± 2.2
Obsession with COVID-19 scale	1.7 ± 1.6

In this study, we hypothesized that (a) depression will be positively related to obsession with COVID-19, (b) viral anxiety will be positively associated with obsession with COVID-19, and (c) reassurance-seeking behavior will at least partially mediate the relationships between depression/viral anxiety and obsession with COVID-19 among medical students.

There was a positive correlation between medical school students experiencing obsession with COVID-19 and depression (rho = 0.29, *p* < 0.001), viral anxiety (rho = 0.60, *p* < 0.01), and coronavirus-related reassurance-seeking behaviors (rho = 0.56, *p* < 0.001). We observed that the hypothesis (a) and (b) was supported by the results. In addition, the PHQ-9 scores were significantly correlated with the CRBS (rho = 0.27, *p* < 0.001), and SAVE-6 (rho = 0.16, *p* = 0.042). The SAVE-6 scale scores significantly correlated with the CRBS scores (rho = 0.38, *p* < 0.001, Table 2). The more severe the degree of depressive symptoms or viral anxiety in medical school students, the more reassurance-seeking behavior they engaged in.

**Table 2:** Spearman's correlation coefficients (rho) of each variables for all participants.

**Variables**	**Age**	**Grade**	**OCS**	**PHQ-9**	**SAVE-6**
Grade	0.80**				
OCS	0.01	0.02			
PHQ-9	−0.02	−0.11	0.29**		
SAVE-6	−0.06	−0.06	0.60**	0.16**	
CRBS	0.06	0.02	0.56**	0.27**	0.38**

*OCS, Obsession with COVID-19 scale; PHQ-9, patient health questionnaire-9; SAVE-6, stress and anxiety to viral epidemics-6 items; CRBS, coronavirus reassurance-seeking behaviors scale;*

* *p < 0.05;*

** *p < 0.01*.

A multiple linear regression analysis was performed to explore whether obsession with COVID-19 among medical students could be expected age, depression, viral anxiety, and reassurance- seeking behavior (Table 3). Obsession with COVID-19 was expected in the PHQ-9 (β = 0.15, *p* = 0.01), SAVE-6 (β= 0.43, *p* < 0.001), and CRBS (β = 0.38, *p* < 0.001) scale scores. Depressive symptoms, viral anxiety, and reassurance-seeking behaviors could explain 49% of COVID-19 obsession among medical school students (adjusted R^2^ = 0.49, F = 39.9, *P* < 0.001). Age was not a significant predictor.

**Table 3:** Linear regression analysis expecting obsession with COVID-19 among medical students.

**Dependent variables**	**Included parameters**	**Beta**	***P*-value**	**Adjusted R^**2**^**	***F, P*-value**
OCS	Age	0.04	0.45	0.49	*F* = 39.9
	PHQ-9	0.15	0.01		*P* < 0.001
	SAVE-6	0.43	<0.001		
	CRBS	0.38	<0.001		

*OCS, Obsession with COVID-19 scale; PHQ-9, Patient Health Questionnaire-9 items; SAVE-6, Stress and Anxiety to Viral Epidemics - 6 items; CRBS, Coronavirus Reassurance-Seeking Behaviors Scale*.

**Figure 1:**
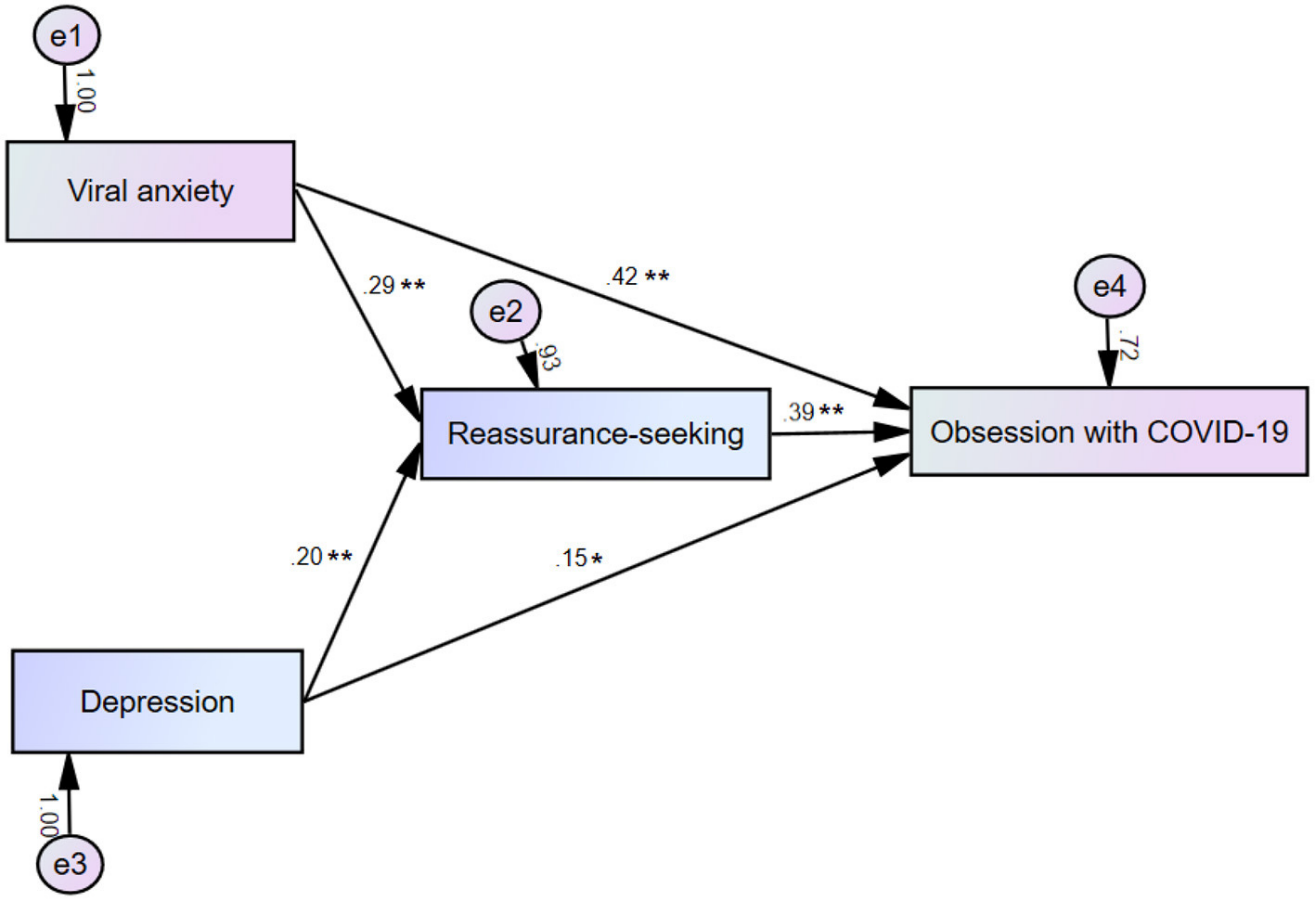
Mediation model showing that the effect of viral anxiety or depression (independent variables) on obsession with coronavirus (outcome) is mediated by reassurance-seeking behavior (mediator). *p < 0.05, **p < 0.01.

Mediation analysis showed that the complete pathway from depression or viral anxiety (independent variable) to reassurance-seeking behavior (mediator) to obsession with COVID-19 (dependent variable) was significant (Table 4). This indicated that reassurance-seeking behavior partially mediated the effects of depression or viral anxiety on obsession with COVID-19 (Figure 1). The total effect of 0.53 for SAVE-6 was decomposed into an indirect effect of 0.11 *(p* < 0.001, CI = 0.05 to 0.18) and a direct effect of 0.42 (p < 0.001, CI = 0.31 to 0.54). For PHQ-9, the total effect of 0.23 is decomposed into an indirect effect of 0.08 (CI = 0.02 to 0.14) and a direct effect of 0.15 (*p* = 0.011, CI = 0.03 to 0.26). The hypothesis (c) was supported by this mediation analysis results.

**Table 4:** The results of direct, indirect, and total effects on mediation analysis.

**Effect**	**Standardized estimate**	**S.E**.	***Z*-value**	***p***	**95% CI**
**Direct effect:** SAVE-6 → OCS PHQ-9 → OCS	0.42 0.15	0.059 0.057	7.157 2.544	<0.001 0.011	0.306 to 0.536 0.034 to 0.259
**Indirect effect:** SAVE-6 → CRBS → OCS PHQ-9 → CRBS → OCS	0.11 0.08	0.034 0.031	3.364 2.538	<0.001 0.011	0.047 to 0.179 0.018 to 0.141
**Component** SAVE-6 → CRBS CRBS → OCS PHQ-9 → CRBS	0.29 0.39 0.20	0.04 0.04 0.04	3.94 6.53 2.76	<0.001 <0.001 0.006	0.07 to 0.21 0.20 to 0.3 8 0.04 to 0.21
**Total effect:** SAVE-6 → OCS PHQ-9 → OCS	0.53 0.23	0.063 0.063	8.459 3.574	<0.001 <0.001	0.410 to 0.658 0.102 to 0.350

*S.E., standard error; CI, confidence interval; SAVE-6, Stress and Anxiety to Viral Epidemics-6 items; PHQ-9, Patient Health Questionnaire-9 items; CRBS, Coronavirus Reassurance-Sseeking Behavior Scale; OCS, Obsession with COVID-19 scale*.

## Discussion

In this study, we investigated whether or not COVID-19 obsession among medical students was a predictor of depression, viral anxiety, and coronavirus reassurance-seeking behavior. The mediation analysis results showed that medical students' viral anxiety and depression affected their obsession with COVID-19, both separately and directly, and their reassurance-seeking behaviors mediated the effect of depression or viral anxiety on obsession with COVID-19.

### Obsession Evaluation During the Pandemic Period

During past epidemics or pandemics, anxiety and obsessive thoughts have been repeatedly reported as a response to threatening public health crises (39, 40). Viral anxiety is helpful in protecting oneself from viral infections during the pandemic, but excessive anxiety and obsessive thoughts may harm mental health. As the COVID-19 pandemic continues for a long period, persistent anxiety and obsessive thoughts must be managed. By using the OCS, we evaluated medical students' maladaptive obsessive thinking about COVID-19, and observed that it was significantly correlated with viral anxiety, depression, and reassurance-seeking behavior. Medical students are highly prone to experience viral anxiety because they constantly find themselves in high-risk environments while conducting clinical clerkship. It was reported that 28.5% of medical students had anxiety in China (41), 28.5% in Peru (42), 25–34.1% in Turkey (43), and 22.6% in Switzerland (44). According to Jennifer et al. it was reported that in the aftermath of COVID-19, medical students were significantly distressed and suffer from psychological burden, which is more severe in 4th grade students (45). A study in India also found that more than 20% of medical students reported depression or anxiety as a result of COVID-19, and more than half of students report excessive academic anxiety (46). However, no previous study which explored the reassurance-seeking behavior or obsession with COVID-19 among medical students was done.

In previous studies, anxiety was measured with “*non-specific*” anxiety measurement tools such as Generalized Anxiety Disorders-7 items, Depression Anxiety Stress Scale-21, or Beck Anxiety Inventory. Since we used the SAVE-6 scale, specifically developed to measure one's anxiety response specific to viral epidemics (28), we can be certain of the influence of medical students' viral anxiety, rather than general anxiety on their obsessive thoughts.

### Obsession With COVID-19 in Relation With Depression, Viral Anxiety, and Reassurance-Seeking Behavior

Individuals who feel anxious may seek assurance to relieve their anxiety and the risk of potential harm (47). Studies on the subjective fear of COVID-19 have shown that while the fear of being sick or death from COVID-19 is prevalent in young patients, hopelessness is seen in the elderly (26, 48). Medical students' viral anxiety may provoke reassurance-seeking behavior, such as checking their body temperature, repetitive hand-washing, or monitoring their physical symptoms during their clinical clerkship. This reassurance-seeking behavior may transiently reduce their anxiety, but this transient relief of anxiety may also strengthen their tendency to seek reassurance. The term “medical student syndrome” (49) was used to reflect the phenomenon of the development of hypochondriacal concerns among medical students about the diseases they are studying. It was studied in relation to their reassurance-seeking behaviors as in the hypochondriasis model. During the COVID-19 pandemic, medical students have increasingly become interested in infectious diseases, and their transmission and prognosis. Hypochondriac fears can provoke reassurance-seeking behavior. Of course, it is not easy to dismiss repetitive hand washing as a reassurance-seeking behavior, as it is also an important prevention measure. Furthermore, “cyberchondria” or excessive online health research associated with increasing levels of health anxiety (50) as a reassurance-seeking behavior, may also lead to negative consequences due to repetitive misleading information from social media (51).

Depression is a mental illness that can have major negative consequences on medical students during the COVID-19 pandemic (52, 53). Perceived stress among Slovak university students increased due to the pandemic, which was positively correlated with their depression (26). Medical students showed significant depression during the pandemic, which predicted their obsession with COVID-19. During the pandemic, the fear of infection, possibility of being assigned the medical field with no prior knowledge about the disease, and online classes being unfavorable to medical care may have been stressful, which may have caused depression. In the present study, depression directly influenced the obsession with COVID-19 patients. Although there is a rare study on the association between depression and obsession with coronavirus in this pandemic, that association was reported while validating the OCS among the general population (36) and special populations such as the police, armed forces (54), and university students (55). Another existing study reported a significant correlation between depression and obsession with COVID-19 among otolaryngologists (56). They must perform potential aerosol-generating procedures such as endoscopy, tracheostomy, or upper airway surgery. The high viral load on the nasal and oropharyngeal mucosa puts otolaryngologists at a higher risk of contracting the disease. Similarly, medical students' depressive symptoms can be associated with their obsession with coronavirus, though we cannot know the causal relationship from the results of our study.

### Reassurance-Seeking Behaviors and Obsession With COVID-19

The role of reassurance-seeking behavior is related to obsessive-compulsive disorder (OCD) (57). It was reported that the severity of obsessive-compulsive symptoms was associated with reassurance-seeking behaviors (58), which reflects that the severity of obsessions can lead one to seek reassurance as a coping strategy. Based on the OCD model (59), compulsive behavior may be a neutralization and safety behavior that can reduce anxiety symptoms caused by obsessions (57). However, repetitive, excessive behaviors or rituals among patients of OCD prevents them from developing tolerance to anxiety due to their obsessive thoughts, leading to persistent OC symptoms (60). During the pandemic, people with excessive viral anxiety were expected to seek reassurance repeatedly (61). Our findings show that, in contrast to the OCD model (invasive thinking causes anxiety and repeated compulsions to relieve anxiety), reassurance-seeking behaviors mediate obsession with COVID-19.

### Reassurance Behaviors Mediate the Effects of Depression or Viral Anxiety on Obsession With COVID-19 in Medical Students

Based on the results of our study, it was found that depression or viral anxiety directly influences obsession with COVID-19, and reassurance-seeking behavior can mediate this association by enhancing the influence of depression or viral anxiety on obsession, rather than reducing the effect. We analyze the reasons behind our results. Firstly, the level of depression or viral anxiety among medical students may be higher than that among the general population. Medical students face harmful situations during their education and clinical clerkship. Although we could not compare the level of viral anxiety between medical students and the general population in this study, higher levels of viral anxiety make them have obsessive thoughts about the virus. Secondly, medical students may indulge in repetitive reassurance-seeking behavior during their clinical clerkship, which may include the need to check their symptoms related to the viral infection, or wash hands to prevent the transmission of the virus. This repetitive reassurance-seeking behavior can increase obsessive thoughts about the virus. Obsession with COVID-19 may be a biologically evolved defense system aiming at safety from harm (62). Medical students should be cautious in preventing viral infections and transmission to patients.

A longitudinal online study of mental health symptoms during the pandemic was conducted in the UK. After the pandemic reached its peak, depressive symptoms and anxiety levels increased; the OC symptoms increased further (63). This was because after the pandemic's peak, the number of infected people started decreasing, and knowledge about the virus and social contacts started increasing due to the relaxations of social distancing norms. However, general uncertainty about the pandemic was still high because of concerns about the second pandemic wave, or the possibility of infection due to the increase in social interaction. With this uncertainty, a coping strategy to obtain reassurance through information-seeking was established, and more information-seeking was sought at a higher OC symptom score (63). As our study was conducted at a time when “living with corona” was considered in Korea, in the same vein, at the beginning of the pandemic, CRB temporarily reduced viral anxiety but excessive reassurance-seeking behavior was established as a maladaptive coping strategy. This can reinforce medical students' OC symptoms.

## Limitations

The present study had some limitations. Firstly, the online survey design of this study could have led to bias. We decided to conduct an online survey rather than face-to-face interviews, to align with the ongoing pandemic situation. This may be related to a lack of reliability of responses. Secondly, only 162 of the 252 UUCM students responded to the survey; the result from a small number of participants from a single medical school cannot be generalized to all medical students across the country. Thirdly, all participants were vaccinated, and more than 70% of them were male, which might have influenced the results as gender influences on the factors studied in this study were not considered. Finally, our study was conducted at a time when we were considering “living with corona.” So it may not be consistent with the results of studies conducted during the early days of the pandemic. Therefore, it is necessary to observe changes in symptoms over time, in future longitudinal studies.

Nevertheless, this study has several strengths. Firstly, as noted above, instead of using the general anxiety scale, we used the anxiety, obsessive-compulsive behavior, and repetitive behavior scales specific to the COVID-19 pandemic. Secondly, our study was conducted with medical students who were vulnerable during the pandemic and who would work as healthcare workers in the future. Thus, the strength of this study lies in its discovery, which states that repetitive behaviors in pursuit of safety led to obsession during the COVID-19 pandemic situation.

## Conclusions

We observed that medical students' viral anxiety and depression separately and directly influenced their obsession with COVID-19, and their reassurance-seeking behavior mediated the influence of depression or viral anxiety on obsession with COVID-19. In this pandemic era, medical school students, who should undergo clinical clerkship and may act as healthcare workers, need to have adaptive coping strategies, and prevent high levels of viral anxiety and repetitive reassurance-seeking behavior for the sake of their mental health.

## Data Availability Statement

The raw data supporting the conclusions of this article will be made available by the authors, without undue reservation.

## Ethics Statement

The studies involving human participants were reviewed and approved by the study protocol was approved by the Institutional Review Board (IRB) of the Asan Medical Center (2021–1438), and obtaining the written informed consent was waived by IRB. Written informed consent for participation was not required for this study in accordance with the national legislation and the institutional requirements.

## Author Contributions

SC, JP, JA, and YH: conceptualization and resources. SC and HK: data curation. SC and JP: formal analysis. JA, YH, and SC: methodology. HK, CK, and JP: writing—original draft. All authors writing—review and editing. All authors contributed to the article and approved the submitted version.

## Conflict of Interest

The authors declare that the research was conducted in the absence of any commercial or financial relationships that could be construed as a potential conflict of interest.

## Publisher's Note

All claims expressed in this article are solely those of the authors and do not necessarily represent those of their affiliated organizations, or those of the publisher, the editors and the reviewers. Any product that may be evaluated in this article, or claim that may be made by its manufacturer, is not guaranteed or endorsed by the publisher.
